# Enhanced Anti-Glioma Efficacy by Borneol Combined With CGKRK-Modified Paclitaxel Self-Assembled Redox-Sensitive Nanoparticles

**DOI:** 10.3389/fphar.2020.00558

**Published:** 2020-04-30

**Authors:** Lingyan Lv, Xinrui Li, Wei Qian, Shennan Li, Yan Jiang, Yaokun Xiong, Jianpei Xu, Wei Lv, Xiaoyan Liu, Yun Chen, Yulin Tang, Hongliang Xin

**Affiliations:** ^1^Department of Pharmacy, The Affiliated Jiangyin Hospital of Southeast University Medical College, Jiangyin, China; ^2^School of Pharmacy, Nanjing Medical University, Nanjing, China; ^3^Department of Pharmacy, Sir Run Run Hospital, Nanjing Medical University, Nanjing, China; ^4^Department of Pharmacy, Zhangjiagang Hospital of Traditional Chinese Medicine, Affiliated Nanjing University of Chinese Medicine, Zhangjiagang, China; ^5^School of Pharmacy, Jiangxi University of Traditional Chinese Medicine, Nanchang, China

**Keywords:** borneol, blood-brain barrier penetration, paclitaxel, redox-responsive nanoparticles, CGKRK peptide

## Abstract

The serious therapeutic obstacles to glioma treatment include poor penetration across the blood-brain barrier (BBB) and low accumulation of therapeutic drugs at tumor sites. In this study, borneol combined with CGKRK peptide (a ligand of the heparan sulfate which overexpress on the glioma cells) modified paclitaxel prodrug self-assembled redox-responsive nanoparticles (CGKRK-PSNPs) were hypothesized to enhance the BBB penetration ability and active tumor targeting efficiency, respectively. The resulting CGKRK-PSNPs possessed a spherical shape with a small particle size (105.61 ± 1.53 nm) and high drug loading for PTX (54.18 ± 1.13%). The drug release behavior proved that CGKRK-PSNPs were highly sensitive to glutathione (GSH) redox environment. The *in vitro* cell experiments suggested that CGKRK-PSNPs significantly increased the cellular uptake and cytotoxicity of U87MG cells, meanwhile CGKRK-PSNPs showed the low cytotoxicity against BCEC cells. Combined with borneol, CGKRK-PSNPs exhibited enhanced transportation across *in vitro* BBB model. In intracranial U87MG glioma-bearing nude mice, the higher accumulation of CGKRK-PSNPs combined with borneol was observed through real-time fluorescence image. Moreover, the *in vivo* anti-glioma results confirmed that CGKRK-PSNPs combined with borneol could improve the anti-glioma efficacy with the prolonged medium survival time (39 days). In conclusion, the collaborative strategy of CGKRK-PSNPs combined with borneol provided a promising drug delivery routine for glioblastoma therapy.

## Introduction

Glioblastoma multiforme (GBM) is the most common and malignant tumor in glioma. It accounts for 50% of primary gliomas in adults with only a 10% probability of 5 years survival (Sasmita et al., 2018). Despite aggressive surgery, radiotherapy, and chemotherapy, the prognosis of GBM has not improved significantly in recent years. The blood-brain barrier (BBB) and blood-brain tumor barrier (BBTB) act as two physical barriers to prevent most chemotherapeutic drugs entering into the brain, which restrict chemotherapy effects (Jain, 2018). In the past few decades, targeted drug delivery vehicles, such as nanoparticle, liposome, and dendrimer, have revolutionized the diagnosis and treatment of glioma (Li and Xie, 2017). However, these nanocarriers still have some drawbacks limiting the clinical applications, for example, the low drug loading, premature release of drug into the blood circulation, without active targeting, and poor permeability to cross the BBB (Hernández-Pedro et al., 2013). The utilization of natural cells as vehicles to encapsulate therapeutic cargoes for drug delivery applications has achieved some progress (Chen et al., 2020). Overall, multidisciplinary technology should be taken together to promote the development of advanced drug delivery systems for cancer therapy (Gao, 2017; Qian et al., 2018).

Borneol (Bor) is widely used in traditional Chinese medicine, which can assist other drugs enter the brain. Studies have reported that Bor could open the intercellular tight junctions and improve the permeation of drugs across the BBB, therefore enhance their concentration in the brain (Guo et al., 2019; Meng et al., 2019). However, there is a great risk for anti-glioma drugs because they will cause serious side effects to normal brain tissue due to lacking of active targeting ability (Han et al., 2018).

CGKRK peptide, which was discovered using *in vivo* phage display technique, was demonstrated it could target tumor cells and tumor neovascular through binding to the specific receptor heparan sulfate (Lv et al., 2016; Griffin et al., 2017; Zhang et al., 2019). It was reported that CGKRK peptide could specifically target tumor vasculature in high-grade glioma and not to normal vessels (Treps, 2018). Agemy et al. (2013) have demonstrated that CGKRK peptide could effectively penetrate tumor cells and tumor blood vessel endothelial cells.

In order to realize precise release of drugs at tumor sites and reduce toxicity to normal brain tissue, tumor microenvironment-responsive drug delivery systems have been arisen widespread attention (for example ROS, pH, redox, and lysosomal enzymes) (Song et al., 2017; Tao and He, 2018; Zhang et al., 2018). The redox-responsive drug carrier is connected by disulfide bond between the carrier and drug molecule, or formed by two block copolymers linked by disulfide bond with drug molecule self-assemble in solvent (Raza et al., 2018). Studies have demonstrated that the disulfide bond is very stable under normal physiological conditions, but it can be cleaved into thiols by GSH (Han et al., 2019; Yu et al., 2020). The concentration of GSH in cancerous cells is about 1,000 times higher than in plasma (Tian et al., 2018; Zhu et al., 2018), and is several times higher than that in normal cells (Liu et al., 2011). In addition, tumor tissue is more reductive than normal tissue due to hypoxia. Therefore, disulfide bond is widely used in GSH trigger system. Our group have studied disulfide bond paclitaxel prodrug (PTX-SS-C_18_) conjugate self-assembled nanoparticles (PSNPs) used as “carrier-free” system to accurately triggered-release PTX in tumor cells by the intracellular GSH (Jiang et al., 2017).

**Figure 1:**
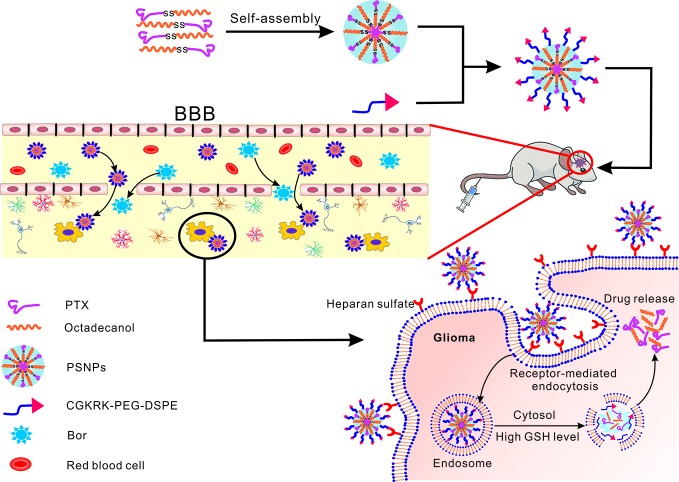
Schematic of borneol combined with CGKRK modified PTX-SS-C_18_ conjugate self-assembled targeted nanoparticles (CGKRK-PSNPs) for glioma treatment.

In this study, we integrated the Bor as a “guide” drug to open the BBB with CGKRK modified PTX-SS-C_18_ conjugate self-assembled active targeted nanoparticles to enhance the anti-glioma efficacy. As shown in **Figure 1**, Bor was intragastrically administrated for 0.5 h in advance to open the tight junctions of BBB reversibly. Then CGKRK-PSNPs were injected *via* tail vein and penetrated across the BBB to accumulate at tumor sites with the assistance of Bor and active glioma target ability. Lastly, CGKRK-PSNPs could be internalized into glioma cells through heparan sulfate-mediated endocytosis, and the PTX was responsively released to the intracellular GSH. The morphology, particle size, and drug release kinetics of CGKRK-PSNPs *in vitro* were all characterized. In addition, cellular uptake and cytotoxic assay were investigated in both BCEC and U87MG cells. Meanwhile, the effect of Bor was evaluated using BBB model *in vitro*. Finally, the intracranial glioma mice model was established to study glioma targeting and anti-glioma efficacy of CGKRK-PSNPs combined with Bor *in vivo*.

## Materials and Methods

### Materials

Paclitaxel (purity > 99.9%) was purchased from Zelang Medical Technology Co., Ltd. (Nanjing, China). DiR (1,1′-dioctadecyl-3,3,3′,3′-tetramethyl indotricarbocyanine iodide) (purity > 95.0%), Dithiodiglycolic acid (purity > 96.0%), 1-Octadecanol (purity > 99.0%), Glutathione(purity > 98.0%) and were obtained from Aladdin Reagent Database Inc. (Shanghai, China). CGKRK peptides (purity > 95%) were purchased from GL Biochem Co., Ltd. (Shanghai, China), Maleimide-PEG2000-DSPE (purity > 99%) and MeO-PEG2000-DSPE (purity > 99%) were purchased from Avanti Polar Lipids, Inc. (Alabama, USA). Bor (purity > 97.0%), glutathione-reduced ethyl ester (purity > 90.0%), and Coumarin-6 (purity ≥ 99%) were provided by Sigma-Aldrich (St. Louis, MO, USA). 3-(4, 5-Dimethylthiazol-2-yl)-2, 5-diphenyltetrazolium bromide (MTT) (purity >99%) and BCA kit were purchased from Beyotime Biotechnology Co., Ltd. (Nantong, China). Penicillin-streptomycin, DMEM medium, fetal bovine serum (FBS), and 0.25% (w/v) trypsin solution were purchased from Gibco BRL (MD, USA). The other chemical reagents were of analytical grade and used as received.

### Preparation and Characterization of CGKRK-PSNPs

CGKRK-PEG-DSPE was synthesized and characterized as follows. Briefly, 10 mg of CGKRK peptide and 4 mg of Mal-PEG_2000_-DSPE were dissolved in 1 ml of PB (0.2 M, pH 7.4) buffer and 1 ml of DMF, respectively. Both of the abovementioned solution were dripped slowly into 8 ml of PB buffer to react with magnetic stirring under nitrogen. The excessive unreacted CGKRK peptide and DMF were removed through dialysis (MWCO 1.0 kDa) against distilled water. Finally, the solution was freeze-dried to obtain CGKRK-PEG-DSPE, which was characterized by ^1^H NMR.

PTX-SS-C_18_, PSNPs, and PEG-PSNPs were synthesized and characterized by previously described method (Jiang et al., 2017). The chemical reaction processes of PTX-SS-C_18_ were shown in **Figure S1**. CGKRK-PSNPs were prepared using ethanol injection method. In brief, 5 mg of PTX-SS-C_18_ was dissolved in 500 μl of anhydrous ethanol and then injected into 10 ml distilled water containing 1 mg CGKRK-PEG-DSPE under 650 rpm stirring for 5 min. After evaporating ethanol at 60°C with a vacuum rotary evaporator, the self-assemble CGKRK-PSNPs were filtrated through 0.45 and 0.22 μm microporous membrane, respectively.

For coumarin-6 or DiR-labeled nanoparticles, both coumarin-6 and DiR were dissolved in anhydrous ethanol using the same method as above.

Transmission electronic microscopy (TEM) (JEOL USA, Wilmington, DE, USA) was used to observe the morphology of nanoparticles. Dynamic light scattering (DLS) (Zs90, Malvern, U.K.) was applied to characterize the particle size and zeta potential of nanoparticles.

### *In Vitro* Reduction-Triggered Drug Release

The *in vitro* release profile of PTX from CGKRK-PSNPs was performed by ultrafiltration centrifugation method. First, PEG-PSNPs and CGKRK-PSNPs were diluted to 4 ml release media, which containing 30 μg of PTX to achieve sink condition. The release medium were phosphate buffer saline (PBS) solutions at pH 7.4 with 1 μM GSH and HAc-NaAc buffer at pH 5.0 with 10 mM GSH containing 0.5% (w/v) Tween-80. The mixtures were kept in 10-ml tubes shaking with a speed of 150 rpm at 37°C. At predetermined time points, the solutions were immediately transferred to ultrafiltration centrifuge tube (MWCO = 30 kDa), centrifuging at 4,000 rpm for 10 min to obtain free PTX. Taking filtrate to measure the concentration of PTX by HPLC analysis.

### *In Vitro* Cellular Uptake

The cellular uptake of PEG-PSNPs and CGKRK-PSNPs was detected qualitatively using fluorescent microscopy. The U87MG cells were implanted at 5 × 10^4^ cells/well in a 24-well plate. After incubation for 24 h, the cells were exposed to coumarin-6-labeled nanoparticles at the coumarin-6 concentrations of 5, 10, and 30 ng/ml for 1 h at 37°C and 4°C, respectively. Afterward, the cells were rinsed with cold PBS thrice and immobilized with 4% formaldehyde for 15 min. Finally, the samples were observed and photographed with fluorescent microscopy (Imager A1, Zeiss, Germany).

HPLC analysis was utilized to further investigate quantitative cell uptake of nanoparticles in U87MG cells. 1 × 10^5^ of U87MG cells per well were inoculated in a 24-well plate. After 24 h, the cells were incubated with PEG-PSNPs and CGKRK-PSNPs at PTX concentrations ranged from 10 to 100 μg/ml for 1 h at 37°C or 4°C, respectively. After washed with cold PBS, the cells were lysed by 400 μl of 1% TritonX-100 per well, and then shook for 10 min. Next, the samples were centrifuged at 8000 rpm for 10 min. In order to determine the total cell protein content, an aliquot of the cell lysate from each well was analyzed using BCA protein assay. The PTX concentration of each well was measured by HPLC.

For the competition assay, after 24 h of cell culture, CGKRK peptide was added to the wells at a concentration of 200 μg/ml at 37°C for 30 min. Next, the cells were washed twice with PBS, and then added the media containing coumarin-6-labeled CGKRK-PSNPs or CGKRK-PSNPs, followed the preceding steps for both qualitative and quantitative assays.

### *In Vitro* Cytotoxicity

The MTT assay was used to evaluate the cytotoxicity of PEG-PSNPs and CGKRK-PSNPs in U87MG and BCEC cells. First, 5 × 10^3^ cells/well of U87MG and BCEC cells were cultured in the 96-well plate for 24 h. Then, the cells were treated with Taxol^®^, PEG-PSNPs and CGKRK-PSNPs at different PTX concentration (0.01, 0.1, 1, 5, 10 μg/ml), respectively. After 48 h, 20 μl/well of MTT (5 mg/ml) reagents were added to incubate another 4 h. Subsequently, the mixture was removed, and each well was supplemented with 200 μl of DMSO shaking in the darkness for 10 min to dissolve formazan crystals completely. Finally, the cell viability was measured using a microplate reader (Thermo Multiskan MK3, USA) with the excitation wavelength at 490 nm.

In order to verify the redox response of drug release of PSNPs in cells, the U87MG and BCEC cells were seeded as described above and pretreated with or without 10 mM glutathione monoester (GSH-OEt). After incubation for 2 h at 37°C, the cells were washed twice with PBS to remove extracellular residual GSH-OEt, and then 1 and 5 μg/ml of different PTX formulations were added into the cells for 48 h. U87MG and BCEC cells without GSH-OEt pretreatment were used as the control, respectively. The cell cytotoxicity was evaluated by the MTT method.

### BBB Penetration *In Vitro*

BCEC cell monolayer was used to establish the BBB model *in vitro*. In briefly, 1×10^5^ BCEC cells were seeded into transwell filters with the polycarbonate membrane of 3 μm pore size for 10 days. The BBB transport model was evaluated by measuring transepithelial electrical resistance (TEER) value. Afterward, 200 μl of coumarin-6-labeled PEG-PSNPs and CGKRK-PSNPs with or without Bor (10 μg/ml) were added into upper compartment, respectively. After incubation for 0.5, 1, 1.5, 2, 2.5, 3, and 4 h, 200-μl samples were taken from basal chamber and supplied with the equal volume of culture medium. The coumarin-6 concentration of sample was determined using microplate reader of multi-wavelength.

### *In Vivo* Imaging Analysis

Four to 5 weeks male Balb/c nude mice weighing approximately 20 g were obtained from BK Lab Animal Ltd. (Shanghai, China). All animal experiments were approved by the laboratory animal ethics committee of Nanjing Medical University. The operational procedures were performed according to protocols evaluated.

The real-time fluorescence imaging analysis was used to evaluate the biodistribution of CGKRK-PSNPs *in vivo*. Intracranial U87MG tumor-bearing mice were established as described previously (Xin et al., 2011; Wang et al., 2014). After 18 days, the glioma-bearing mice were randomly dived into 4 groups. The mice in groups 2 and 4 were given 100 μl of DiR-labeled PEG-PSNPs and CGKRK-PSNPs *via* tail vein, respectively. For groups 1 and 3, besides PEG-PSNPs and CGKRK-PSNPs, 25 mg/kg Bor solution was administrated intragastrically before 0.5 h. Next, the mice were scanned using an *in vivo* imaging system (Caliper, USA) at 4 h to acquire the fluorescent images. Afterward, all mice were sacrificed, and their organs were harvested for *ex vivo* fluorescent imaging.

### *In Vivo* Anti-Glioma Efficacy

To evaluate the *in vivo* anti-glioma activity of different PTX formulations, intracranial glioma bearing nude mice were randomly divided into six groups and administered with saline, Taxol^®^, PEG-PSNPs, PEG-PSNPs with Bor, CGKRK-PSNPs, and CGKRK-PSNPs with Bor every other day for four times at a PTX dose of 10 mg/kg, respectively. For nanoparticles with Bor group, the mice were orally administrated with 25 mg/kg Bor 0.5 h before intravenous injection of nanoparticles suspension. Next, the survival time of each mice was recorded to plot Kaplan-Meier survival curves.

### Statistical Analysis

All the results were expressed as mean± standard deviation (SD). One-way ANOVA was utilized for statistical evaluation. Statistical analysis was performed with SPSS 20.0 software. Differences were considered significant when *P< 0.05, **P< 0.01, ***P < 0.001, respectively.

## Results and Discussion

### Characterization of Nanoparticles

**Figure 2:**
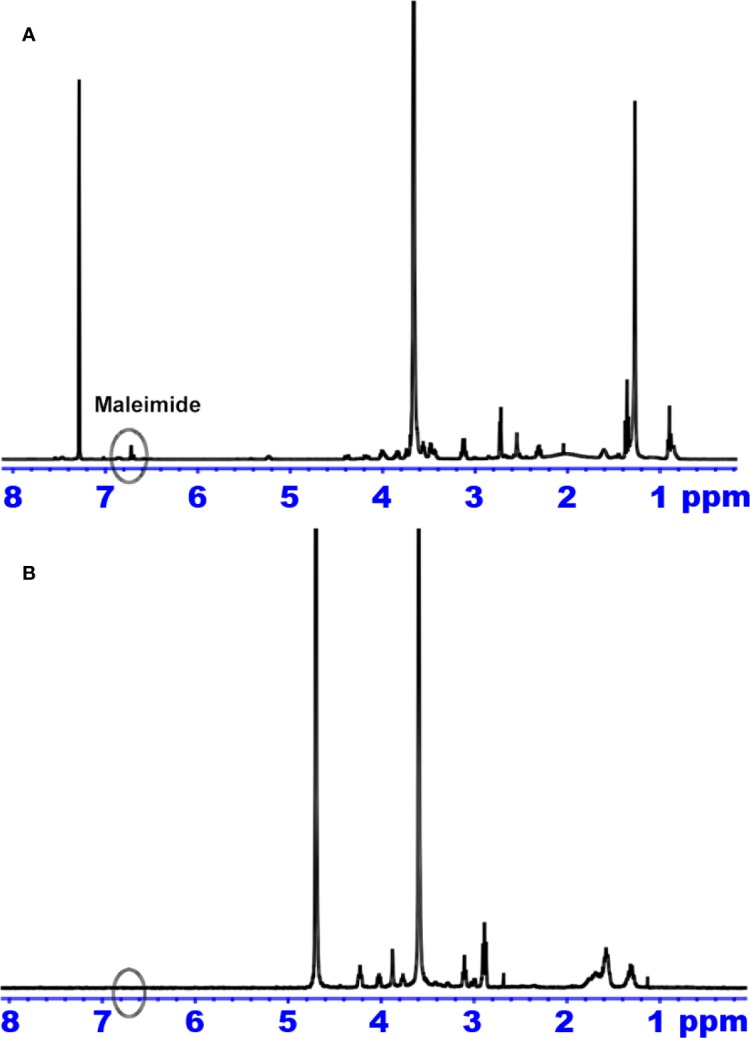
^1^H NMR spectrum of Mal-PEG_2000_-DSPE **(A)** and CGKRK-PEG-DSPE **(B)**.

The structures of Mal-PEG_2000_-DSPE and CGKRK-PEG-DSPE were determined by ^1^H NMR (**Figure 2**). The methylene protons of PEG in Mal-PEG_2000_-DSPE was at 3.6 ppm (**Figure 2A**), and the maleimide group has a characteristic peak at 6.7 ppm (**Figure 2A**) before reaction. However, after reaction with CGKRK peptide, the maleimide peak (δ 6.7) disappeared in the ^1^H NMR spectra of CGKRK-PEG-DSPE (**Figure 2B**), whereas the PEG segment was still presented at 3.6 ppm (**Figure 2B**). It suggested that CGKRK peptide was conjugated with Mal-PEG_2000_-DSPE.

PTX-SS-C_18_ compound was synthesized and characterized according to our previous study (Jiang et al., 2017). The CGKRK-PSNPs were prepared *via* ethanol injection method. The physical characterizations of nanoparticles including particle size, polymer dispersion index (PDI), zeta potential, and loading capacity (LC) were shown in **Table 1**. The mean particle size of PEG-PSNPs and CGKRK-PSNPs were 96.57 ± 1.25 nm and 105.61 ± 1.53 nm, respectively, with a narrow distribution (PDI < 0.2). Typically, nanoparticles with a particle size of 20 to 200 nm could passively extravasate and accumulate at malignant sites through the enhanced permeability and retention (EPR) effects (Deshantri et al., 2018). After modified with CGKRK peptide, the particles size had a slight increase but still close to 100 nm, which was beneficial for tumor drug delivery. All the nanoparticles showed negative zeta potential (about −30 mV). The LC of PEG-PSNPs and CGKRK-PSNPs were 55.41 ± 1.27% and 54.18 ± 1.13%, respectively, which was superior to traditional PTX nanoformulations with less than 10% (w/w) (Zhang et al., 2013; Nehate et al., 2014).

**Table 1:** Characterizations of various nanoparticles.

	PEG-PSNPs	CGKRK-PSNPs
Particle size (nm)	96.57 ± 1.25	105.61 ± 1.53
Polydispersity index (PDI)	0.14 ± 0.05	0.16 ± 0.06
Zeta potential (-mV)	−30.6 ± 1.32	−28.4 ± 1.21
Loading capacity (LC %)	55.41 ± 1.27	54.18 ± 1.13

Data are represented with mean ± SD (n=3).

**Figure 3:**
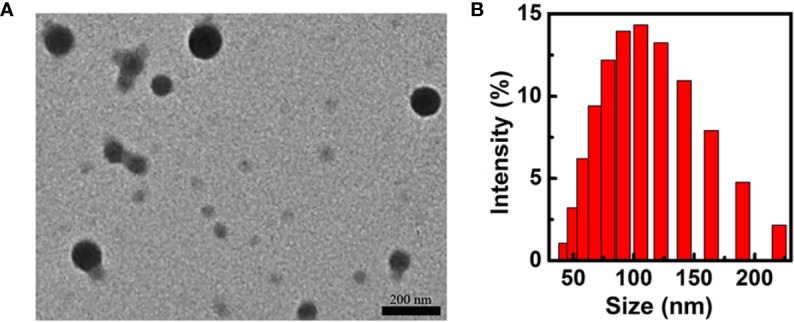
TEM images **(A)** and particle size and size distribution **(B)** of CGKRK-PSNPs.

The morphology of CGKRK-PSNPs was observed by TEM (**Figure 3A**). CGKRK-PSNPs showed spherical shape, and the diameter was about 100 nm, which was in line with the size distribution (**Figure 3B**).

### *In Vitro* Reduction-Triggered Drug Release

**Figure 4:**
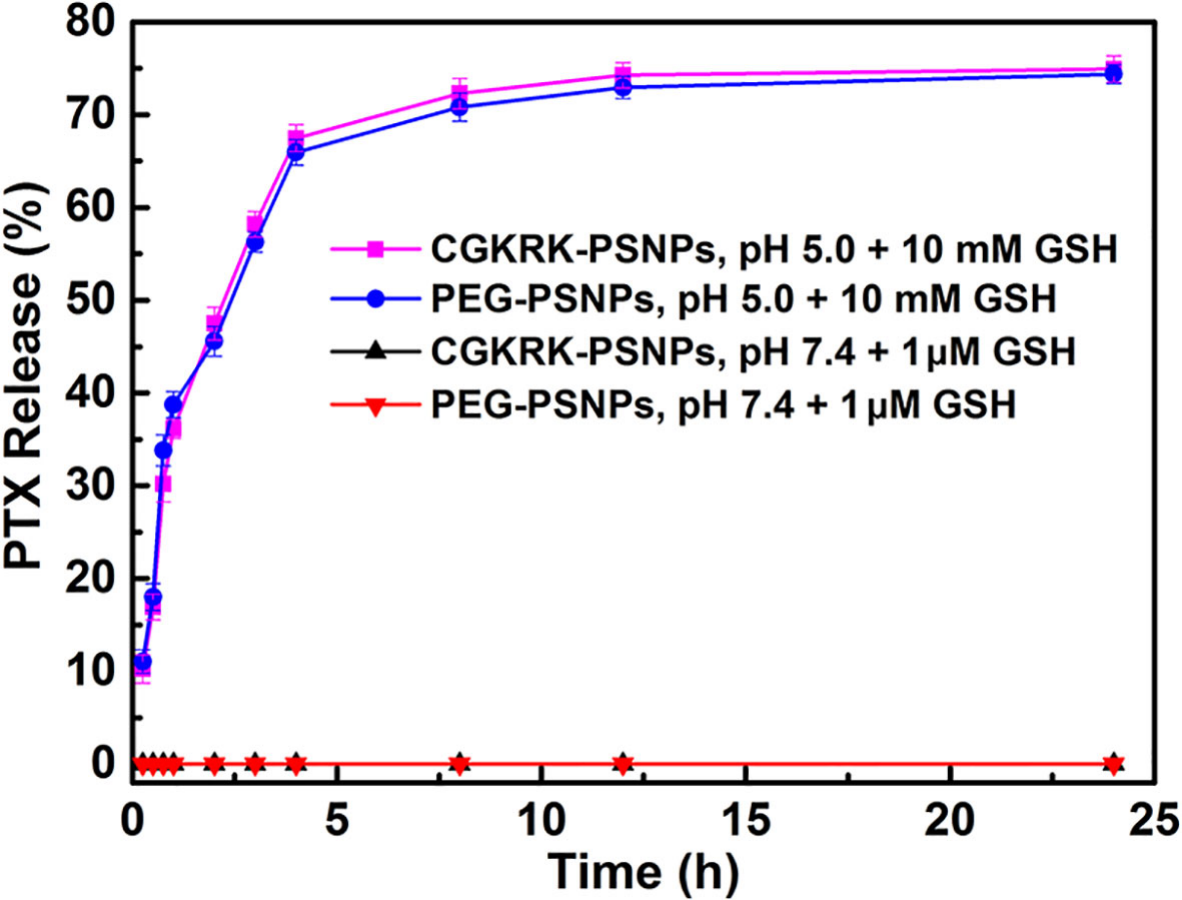
Reduction-triggered PTX release from CGKRK-PSNPs with 1 μM GSH or 10 mM GSH (n = 3).

In previous reports, the intracellular GSH concentration in tumor cells is about 2 to 10 mM, while in the plasma is almost 1 to 2 μM (Maggini et al., 2016). In this study, 10 mM GSH was used to mimic the intracellular redox condition. The *in vitro* PTX release behavior was shown in **Figure 4**, when exposed to 1 μM GSH (pH = 7.4) solution, it showed that the nanoparticles remained stable with no PTX release over 24 h. By contrast, when the GSH concentration was adjusted to 10 mM, more than 50% of PTX was released before 5 h. After 24 h, PEG-PSNPs and CGKRK-PSNPs showed the cumulative release of PTX were 74.37% and 74.92%, respectively. These results suggested that CGKRK-PSNPs were highly sensitive to redox environment, which indicating the disulfide bond could rapidly be cleaved in cancer cells to release PTX. The reduction-responsive PTX-SS-C_18_ conjugate self-assembled targeted nanoparticles provided a great potential to improve the anti-tumor effect.

### *In Vitro* Cellular Uptake

**Figure 5:**
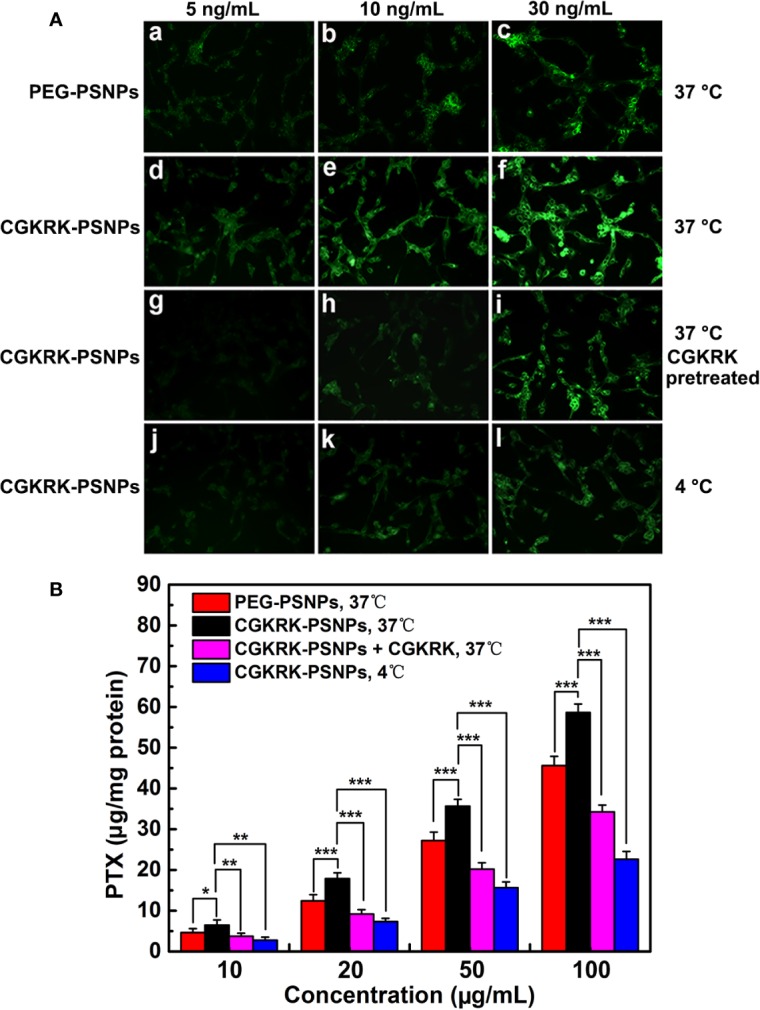
Cellular uptake of coumarin-6-labeled PEG-PSNPs **(a**–**c)** and CGKRK-PSNPs **(d–l)** at 37°C **(a–i)** and 4°C **(j–l)** after incubation for 1 h at the coumarin-6 concentration of 5 **(a, d, g, j)**, 10 **(b, e, h, k)**, and 30 ng/ml **(c, f, i, l)** in U87MG and CGKRK pretreated U87MG cells **(g–i)** was examined by fluorescent microscopy **(A)**. Original magnification: ×20. U87MG uptake of PEG-PSNPs and CGKRK-PSNPs at different conditions after incubation for 1 h at the PTX concentrations from 10 to 100 μg/ml (n = 3) **(B)**. ^***^P < 0.001, ^**^P < 0.01, *P < 0.05.

The cellular uptake assay was performed qualitatively by fluorescent microscopy with coumarin-6 as the fluorescence probe. As shown in **Figure 5A**, the fluorescence intensity of U87MG cells treated with PEG-PSNPs and CGKRK-PSNPs was increased with the increasing concentration of coumarin-6 ranging from 5 to 30 ng/ml, suggesting concentration-dependent cellular uptake of nanoparticles. From **Figures 5Aa–f**, it was shown that CGKRK-PSNPs exhibited stronger fluorescence intensity as compared to that of PEG-PSNPs at all the detected concentrations after 1 h incubation. Besides, the competition assay showed that CGKRK-PSNPs group that pre-incubated with free CGKRK peptide exhibited much weaker fluorescence intensity than that of untreated ones at the same concentration of coumarin-6, respectively (**Figures 5Ag–i**). Moreover, the fluorescence intensity of CGKRK-PSNPs decreased when the incubation temperature dropped from 37°C to 4°C (**Figures 5Aj–l**).

The quantitative results were fully consistent with qualitative fluorescence intensity (**Figure 5B**). The cellular association of CGKRK-PSNPs was 1.4, 1.44, 1.31, and 1.29 folds higher than that of PEG-PSNPs on U87MG cells at the PTX concentration of 10, 20, 50, and 100 μg/ml at 37°C, respectively. In competition assay, the cellular uptake of CGKRK-PSNPs was 1.74, 1.95, 1.76, and 1.71 folds higher than that of pretreated with free CGKRK peptide at 37°C, respectively. In addition, the cellular uptake of CGKRK-PSNPs at 37°C was 2.33, 2.44, 2.28, and 2.59 folds higher than that of 4°C, respectively.

Based on qualitative and quantitative experiment results, it indicated that CGKRK peptide acted as a positive targeting role in CGKRK-PSNPs cellular uptake through heparan sulfate receptor-mediated endocytosis in U87MG cells.

### *In Vitro* Cytotoxicity

**Figure 6:**
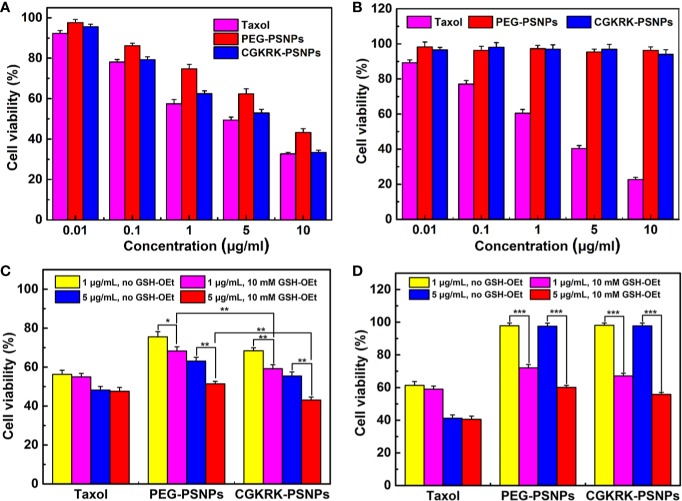
Cytotoxicity of Taxol^®^, PEG-PSNPs, and CGKRK-PSNPs in U87MG cells **(A)** and BCEC cells **(B)** after incubation for 48 h (n = 6). Viability of GSH-OEt pretreated or non-pretreated U87MG cells **(C)** and BCEC cells **(D)** incubated with Taxol^®^, PEG-PSNPs and CGKRK-PSNPs for 48 h. ^***^P < 0.001, ^**^P < 0.01, *P < 0.05.

The *in vitro* anti-proliferation of different PTX formulations against U87MG and BCEC cells was evaluated using the MTT assay. The results (**Figure 6A**) showed that all the PTX formulations inhibited U87MG cells viability in a concentration dependent manner with the increasing concentration of PTX ranging from 0.01 to 10 μg/ml. In line with the cellular uptake results, CGKRK-PSNPs displayed significantly stronger cytotoxicity as compared to PEG-PSNPs. As shown in **Figures 6A, B**, Taxol^®^ exhibited much lower cell viability than nanoparticles, because they could passively transport into cells and diffused to cytosol rapidly (Tian et al., 2018), whereas nanoparticles needed to be internalized by endocytosis and then the disulfide bond would be cleaved to release PTX under high concentration of GSH (Feng et al., 2018). In BCEC cells (**Figure 6B**), PEG-PSNPs and CGKRK-PSNPs displayed negligible cytotoxicity at all the detected concentrations, which suggested that the GSH concentration was too low to cleave disulfide bond in normal cells. These results implied that CGKRK-PSNPs could improve the cellular uptake *via* receptor-mediated endocytosis and then further enhance the cytotoxicity to tumor cells, meanwhile the redox-responsive characteristic of CGKRK-PSNPs realized precise release of PTX in tumor cells, avoiding the cytotoxic side effects to normal cells.

To further investigate whether PTX release from nanoparticles triggered by different intracellular GSH concentration, exogenous GSH was artificially added into the cell as external enhancers to increase the intracellular concentration of GSH (Zhang et al., 2016; Wang et al., 2017). It was reported that GSH-OEt could generate the high intracellular concentration of GSH by ethyl ester hydrolyzation in cytoplasm (Liu et al., 2011). The cytotoxicity of the redox-responsive nanoparticles was further analyzed by MTT assay against U87MG and BCEC cells. Before the addition of various PTX formulations, the cells were firstly pretreated with 10 mM GSH-OEt for 2 h. It proved that GSH-OEt did not affect the viability and endocytosis ability of the cells at the tested concentration (Zhang et al., 2016). Cells without pretreatment were used as the control. As shown in **Figures 6C, D**, PEG-PSNPs and CGKRK-PSNPs exhibited significant higher inhibitory effect on both U87MG and BCEC cells pretreated by GSH-OEt when compared with the control cells. It was noted that CGKRK-PSNPs showed significant cytotoxicity than PEG-PSNPs to the pretreated U87MG cells (**Figure 6C**). At the same time, the proliferation of U87MG or BCEC cells incubated with Taxol^®^ was not affected after adding GSH-OEt to the cell culture media at PTX dose of 1 and 5 μg/ml. These results demonstrate that the GSH concentration in tumor cells can accelerate PTX release from PSNPs, and then increase the cytoplasm uptake of PTX, afterward enhance proliferation inhibition to tumor cells. Therefore, it indicates that CGKRK-PSNP is a promising site-specific delivery carrier for tumor treatment.

### BBB Penetration *In Vitro*

**Figure 7:**
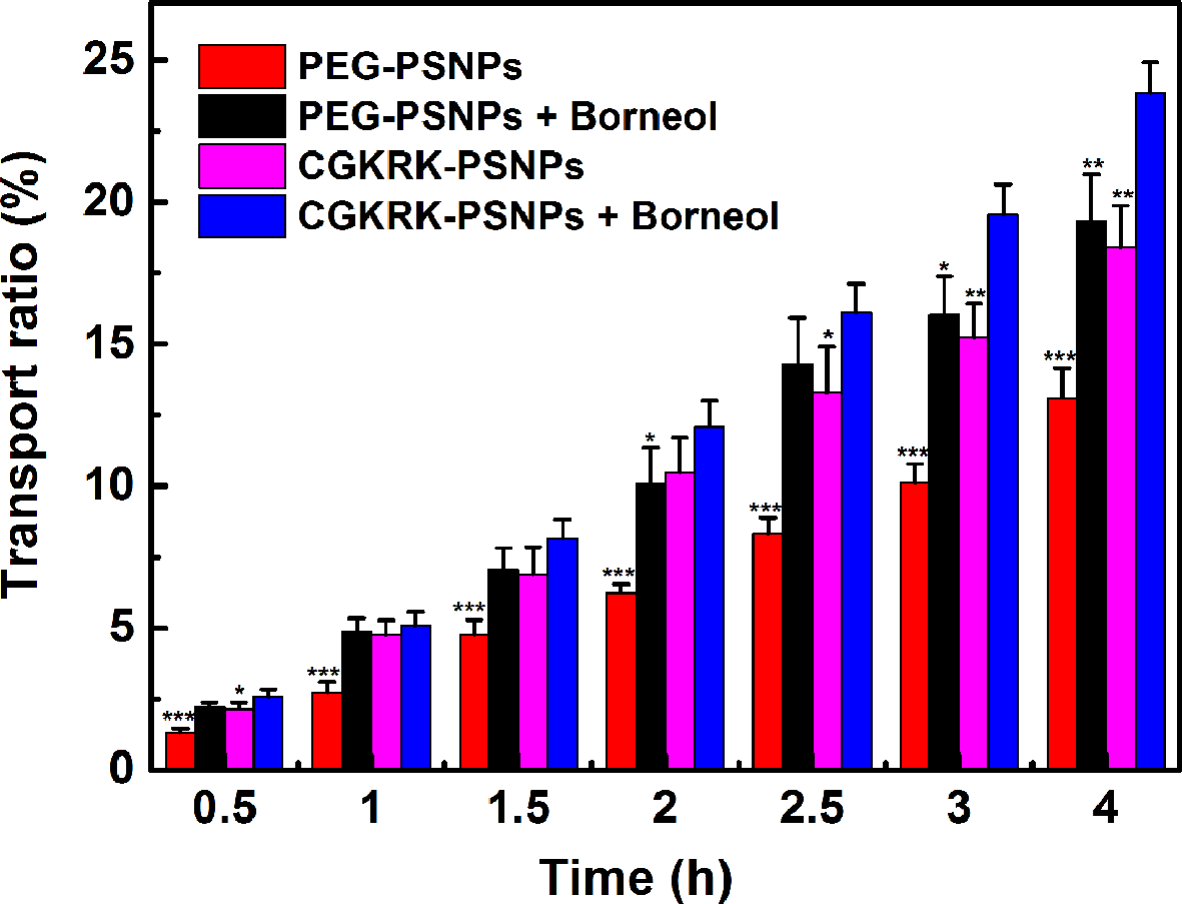
The transport ratios of PEG-PSNPs and CGKRK-PSNPs (with or without borneol) across the BBB model *in vitro*. ^***^P < 0.001, ^**^P < 0.01, ^*^P < 0.05 significantly lower than that of CGKRK-PSNPs with borneol.

The BBB maintains the brain microenvironment homeostasis due to its unique structure and cellular composition, which consists of capillary endothelium with its tight junctions, basal lamina, the end-feet of astrocytes and pericytes (Seo et al., 2019). This special physical barrier prevents most pharmaceutical anti-tumor drugs from entering the brain. In this study, we used BCEC cells to establish the *in vitro* BBB model to investigate the penetration efficiency of CGKRK-PSNPs with the assistance of Bor. As shown in **Figure 7**, the BBB permeability of CGKRK-PSNPs combined with Bor was significantly higher than the other groups after incubated for 3 h. The transport behavior of different nanoparticles displayed a time-dependent profile. After 4 h incubation, the transportation ratios were 19.33% and 13.11% for PEG-PSNPs (with or without Bor), 23.85% and 18.38% for CGKRK-PSNPs (with or without Bor), respectively. The results suggested that Bor could enhance the migration efficiency of both PEG-PSNPs and CGKRK-PSNPs penetrating the *in vitro* BBB. At all the determined time points, the permeability of CGKRK-PSNPs was higher than PEG-PSNPs, which might be due to BCEC as one kind of vascular endothelial cells, there was also a certain expression of heparin sulfate receptor on surface.

### *In Vivo* Imaging Analysis

**Figure 8:**
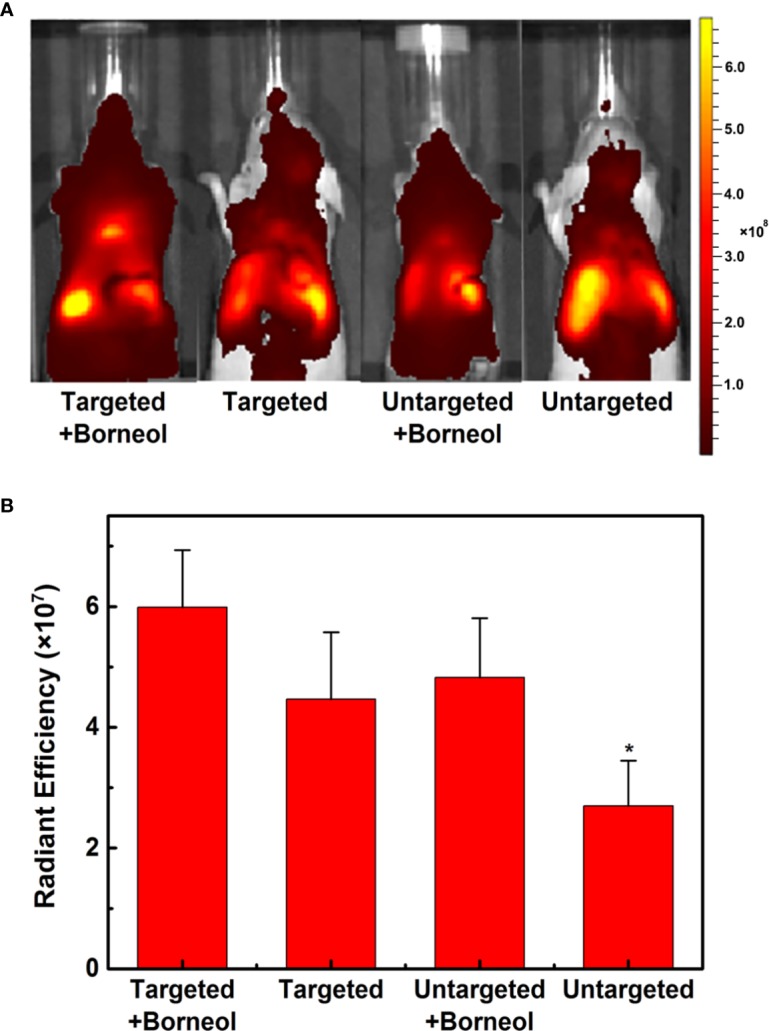
*In vivo* fluorescence imaging of U87MG glioma-bearing nude mice after administration with DiR-labeled CGKRK-PSNPs with borneol, DiR-labeled CGKRK-PSNPs, DiR-labeled PEG-PSNPs with borneol, and DiR-labeled PEG-PSNPs **(A)**. The corresponding semi-quantitative radiant efficiency of brains **(B)**. ^*^P < 0.05 significantly lower than that of CGKRK-PSNPs with borneol.

In order to estimate the brain targeting capability of CGKRK-PSNPs combined with Bor, U87MG glioma-bearing nude mice were imaged using DiR as the fluorescent marker. As shown in **Figure 8A**, the fluorescence intensity of CGKRK-PSNPs and PEG-PSNPs with Bor was higher compared with that without Bor, respectively, which indicted that Bor could improve the BBB penetration. When combined with Bor, the fluorescence signal of CGKRK-PSNPs group was slightly higher than PEG-PSNPs group. In addition, the images of *ex vivo* brains and the semi-quantitative results (**Figures S2** and **8B**) also confirmed that CGKRK-PSNPs combined with Bor exhibited the highest accumulation than other three groups at the glioma sections. Taken together, the enhanced glioma site distribution of CGKRK-PSNPs combined with Bor was attributed to the CGKRK peptide-mediated endocytosis and Bor for BBB penetration enhancement.

### *In Vivo* Anti-Glioma Efficacy

**Figure 9:**
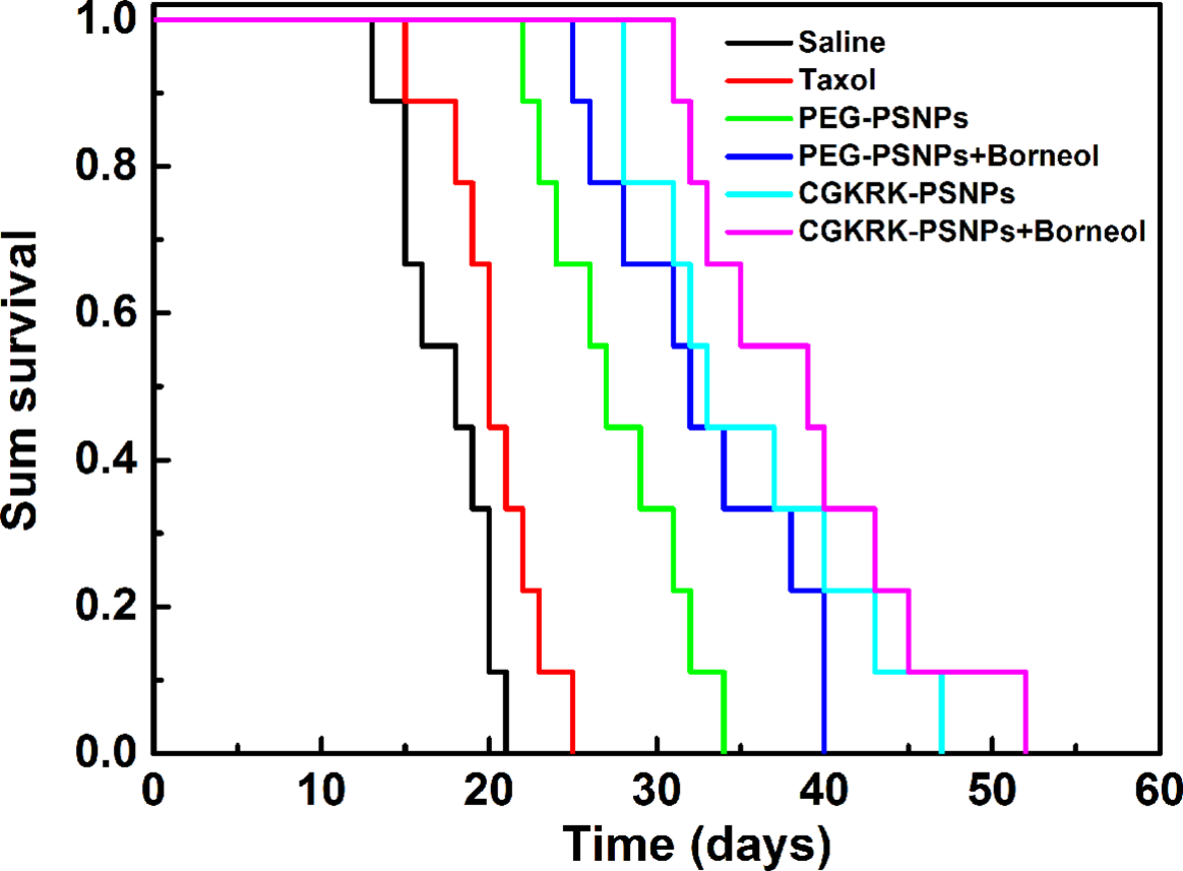
Kaplan-Meier survival curve of U87MG glioma-bearing mice treated with different PTX formulations at a dose of 10mg/kg PTX on day 2, 4, 6, and 8 post implantation (n = 9).

The anti-glioma efficacy of CGKRK-PSNPs combined with Bor was investigated on intracranial U87MG glioma mice models. As shown in **Figure 9** and **Table 2**, the medium survival time of mice treated with CGKRK-PSNPs combined with Bor (39 days) was longer when compared to saline (18 days, *P* < 0.001), Taxol^®^ (20 days, *P* < 0.001), PEG-PSNPs (27 days, *P* < 0.001), PEG-PSNPs with Bor (32 days) and CGKRK-PSNPs (33 days). These results confirmed that CGKRK-PSNPs combined with Bor had great advantage in improving anti-glioma efficacy.

**Table 2:** *In vivo* anti-glioma efficacy of different PTX formulations on intracranial U87MG glioma mice model (n = 9).

Groups	Dose (mg/kg)	MST^a^ (days)	Median (days)	Compare with saline^b^	Compare with Taxol^b^	Compare with PEG-PSNPs^b^
Saline	―	17.4 ± 0.9	18	―	―	―
Taxol	10	20.3 ± 1.0	20	*	―	―
PEG-PSNPs	10	27.6 ± 1.4	27	***	***	―
PEG-PSNPs +borneol	10	32.7 ± 1.9	32	***	***	*
CGKRK-PSNPs	10	35.4 ± 2.2	33	***	***	**
CGKRK-PSNPs +borneol	10	38.9 ± 2.3	39	***	***	***

a MST, mean survival time.

b ^*^P < 0.05, ^**^P < 0.01, ^***^P < 0.001 of log-rank analysis.

## Conclusions

In summary, CGKRK peptide modified redox-sensitivity nanoparticles were successfully developed in this study. *In vitro* release study showed that CGKEK-PSNPs kept stable under physiological conditions and disassembled rapidly with high concentration of GSH. Compared with PEG-PSNPs, CGKEK-PSNPs displayed enhanced cellular uptake and cytotoxicity against U87MG cells. Nevertheless, both of them showed negligible cytotoxicity on BCEC cells. *In vitro* BBB model, CGKEK-PSNPs combined with Bor significantly improved the BBB penetration ability. Furthermore, CGKEK-PSNPs combined with Bor exhibited higher accumulation at the glioma site and extended survival time in U87MG glioma-bearing mice. In conclusion, the strategy that combined Bor with receptor-mediated redox-responsive drug delivery system displayed a great potential for glioma treatment.

## Data Availability Statement

The datasets generated for this study are available on request to the corresponding authors.

## Ethics Statement

The animal study was reviewed and approved by Nanjing Medical University.

## Author Contributions

YC, YT, and HX designed the experiments. LL, XinL, and WQ performed the experiments. LL and HX wrote the main manuscript. SL, YJ, and YX analyzed the data. JX, WL, and XiaL prepared the figures and tables. All authors reviewed the manuscript.

## Funding

This work was supported by the grants from National Natural Science Foundation of China (81973255), Six Talent Peaks Project of Jiangsu Province (SWYY-051), the grants from the Suzhou Science and Technology Plan Project (SYSD2018219), Science and Technology Development Foundation of Nanjing Medical University (2017NJMU216) and Science and Technology Plan Project of Jiangxi Province Education Department (GJJ170736).

## Conflict of Interest

The authors declare that the research was conducted in the absence of any commercial or financial relationships that could be construed as a potential conflict of interest.
