# Supplementary material for: Enhanced Anti-Glioma Efficacy by Borneol Combined With CGKRK-Modified Paclitaxel Self-Assembled Redox-Sensitive Nanoparticles
Source: Front Pharmacol. 2020 Apr 30;11:558. doi: 10.3389/fphar.2020.00558 (PMC7203528; doi:10.3389/fphar.2020.00558)
Supplement: Supplementary file 1 [file DataSheet_1.doc]

**Supporting Information**

**Enhanced anti-glioma efficacy by borneol combined with CGKRK-modified paclitaxel self-assembled redox-sensitive nanoparticles**

Lingyan Lv^1,2#^, Xinrui Li^2,3#^, Wei Qian^4#^, Shennan Li^2^, Yan Jiang^2^, Yaokun Xiong^5^, Jianpei Xu^2^, Wei Lv^1^, Xiaoyan Liu^1^, Yun Chen^2^*, Yulin Tang^3^*, Hongliang Xin^2^*

^1^ Department of Pharmacy, The Affiliated Jiangyin Hospital of Southeast University Medical College, Jiangyin, China

^2^ School of Pharmacy, Nanjing Medical University, Nanjing, China

^3^ Sir Run Run Hospital, Nanjing Medical University, Nanjing, China

^4^ Department of Pharmacy, Zhangjiagang Hospital of Traditional Chinese Medicine, Affiliated Nanjing University of Chinese Medicine, Zhangjiagang 215600, China ^5^ School of Pharmacy, Jiangxi University of Traditional Chinese Medicine, Nanchang, China

^#^ These authors have contributed equally to this work

***Correspondence:** :

Hongliang Xin (E-mail: [xhl@njmu.edu.cn](mailto:xhl@njmu.edu.cn));

Yulin Tang (E-mail: ylt1964@sina.com)

Yun Chen (E-mail: ychen@njmu.edu.cn)


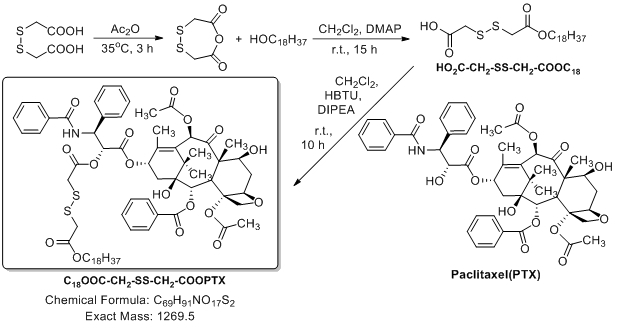


**Figure S1.** The chemical reaction processes of PTX-SS-C_18_.

**
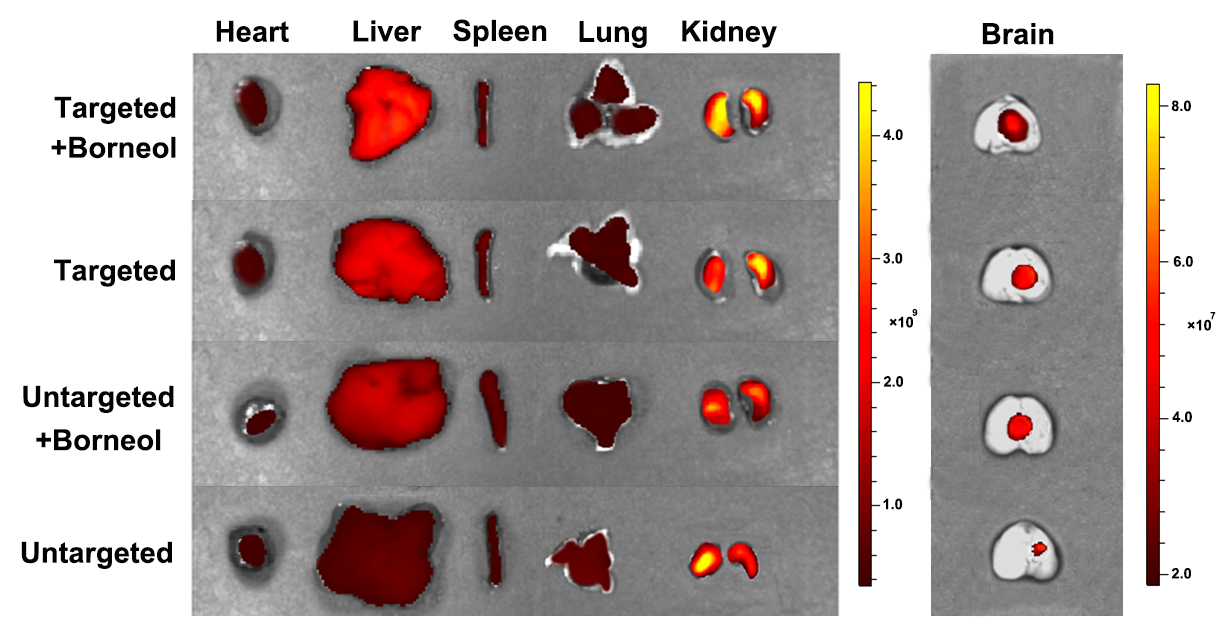
**

**Figure S2.** *Ex vivo* fluorescence imaging of brains and organs at 4 h. .
